# Modulation of the pre-metastatic bone niche: molecular changes mediated by bone-homing prostate cancer extracellular vesicles

**DOI:** 10.3389/fcell.2024.1354606

**Published:** 2024-02-22

**Authors:** Thomas J. Brown, Catrin S. Rutland, Katie K. Choi, Feng Tse, Mandy J. Peffers, Nigel P. Mongan, Kenton P. Arkill, Alison Ritchie, Philip A. Clarke, Hari Ratan, Cinzia Allegrucci, Anna M. Grabowska, Victoria James

**Affiliations:** ^1^ Faculty of Medicine and Health Sciences, School of Veterinary Medicine and Science, University of Nottingham, Sutton Bonington Campus, Loughborough, United Kingdom; ^2^ Faculty of Medicine and Health Sciences, School of Medicine, Biodiscovery Institute, University of Nottingham, Nottingham, United Kingdom; ^3^ Institute of Ageing and Chronic Disease, Liverpool, United Kingdom; ^4^ Department of Pharmacology, Weill Cornell Medicine, New York, NY, United States

**Keywords:** extracellular vesicles, prostate cancer, bone metastasis, pre-metastatic niche, osteocytes

## Abstract

Prostate cancer (PCa) is a leading male malignancy worldwide, often progressing to bone metastasis, with limited curative options. Extracellular vesicles (EVs) have emerged as key players in cancer communication and metastasis, promoting the formation of supportive microenvironments in distant sites. Our previous studies have highlighted the role of PCa EVs in modulating osteoblasts and facilitating tumor progression. However, the early pre-metastatic changes induced by PCa EVs within the bone microenvironment remain poorly understood. To investigate the early effects of repeated exposure to PCa EVs *in vivo*, mimicking EVs being shed from the primary tumor, PCa EVs isolated from cell line PC3MLuc2a were fluorescently labelled and repeatedly administered via tail vein injection to adult CD1 NuNu male mice for a period of 4 weeks. *In vivo* imagining, histological analysis and gene expression profiling were performed to assess the impact of PCa EVs on the bone microenvironment. We demonstrate for the first time that PCa EVs home to both bone and lymph nodes following repeated exposures. Furthermore, the accumulation of EVs within the bone leads to distinct molecular changes indicative of disrupted bone homeostasis (e.g., changes to signaling pathways such as Paxillin *p* = 0.0163, Estrogen Receptor *p* = 0.0271, RHOA *p* = 0.0287, Ribonucleotide reductase *p* = 0.0307 and ERK/MAPK *p* = 0.0299). Changes in key regulators of these pathways were confirmed *in vitro* on human osteoblasts. In addition, our data compares the known gene signature of osteocytes and demonstrates a high proportion of overlap (52.2%), suggesting a potential role for this cell type in response to PCa EV exposure. No changes in bone histology or immunohistochemistry were detected, indicating that PCa EV mediated changes were induced at the molecular level. This study provides novel insights into the alterations induced by PCa EVs on the bone microenvironment. The observed molecular changes indicate changes in key pathways and suggest a role for osteocytes in these EV mediated early changes to bone. Further research to understand these early events may aid in the development of targeted interventions to disrupt the metastatic cascade in PCa.

## Graphical Abstract

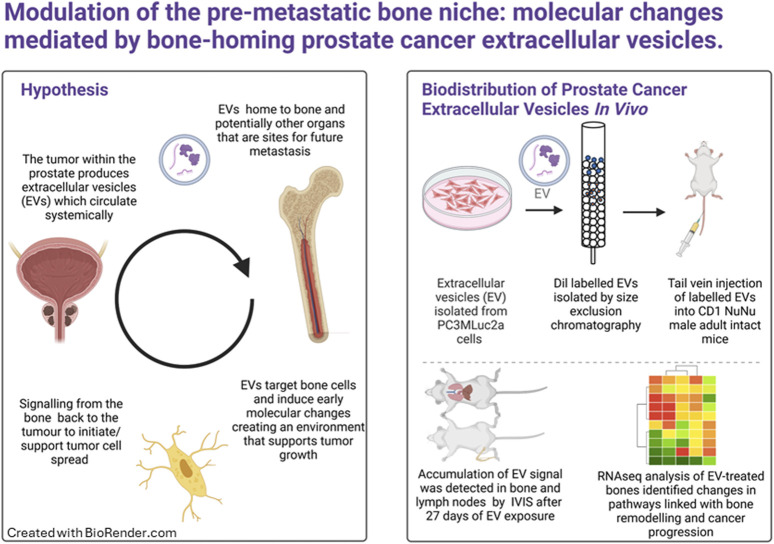

## 1 Introduction

Prostate cancer (PCa) is the most diagnosed male malignancy worldwide, with >1.4 million cases in 2020 (Sung et al., 2021). Progression to bone metastasis occurs in up to 80% of advanced PCa cases (Cancer Research UK, 2023). There remains no curative therapy for metastatic PCa and treatment options at this stage focus on palliative care, with 5-year survival rates at a low of ∼30% (Cancer Research UK, 2023). The axial skeleton is most frequently affected by bone metastases, resulting in serious skeletal-related events (SREs) such as fracture, spinal cord compression (SCC) and hypercalcemia, reducing quality of life and worsening survival (Coleman et al., 2020).

Compelling evidence supports the concept of tumor-secreted factors, such as extracellular vesicles (EVs), as mediators of communication which promote the metastatic process (Jung et al., 2009; Grange et al., 2011; Hood et al., 2011; Rana et al., 2013; Suetsugu et al., 2013; Costa-Silva et al., 2015; Hoshino et al., 2015; Wang et al., 2015; Zomer et al., 2015; Liu et al., 2016; Probert et al., 2019). For example, the cargo of cancer derived EVs has been shown to mediate cancer associated fibroblast (CAF) formation, migration of endothelial cells and enhance angiogenesis, thereby promoting the formation of distant microenvironments supportive of cancer colonization (Naito et al., 2017). The RNA cargo of melanoma secreted EVs were found to result in chemokine secretion and spontaneous lung metastasis in TLR3^−/−^ knockout mice (Liu et al., 2016). Similarly, EVs derived from PCa cell lines have been shown to mediate the differentiation of fibroblasts to myofibroblasts via transforming growth factor-beta (TGF-β) and modulation of osteoblasts to support tumor progression (Webber et al., 2010; Probert et al., 2019).

The term “EVs” describes any type of lipid bilayer bound vesicle released into the extracellular space. Sub-populations of EVs can be classified based on biogenesis, but they also differ in their molecular content, membrane composition, and specific functions. EVs have historically been sub-classified as exosomes, microvesicles and apoptotic bodies. Exosomes are produced by inward budding of the endosomal membrane, with a size range of approximately 30–150 nm, are thought to be subject to regulated cargo loading with a functional role in cell–cell communication (Thery et al., 2009; Colombo et al., 2014). Microvesicles are formed from the outward budding of the plasma membrane and have a broader size range. While microvesicles have been considered to play a role in evacuating waste/unwanted products from cells, more recent reports support a role in cell-to-cell signaling (Heijnen et al., 1999; Kalra et al., 2012).

EVs carry a cargo of genetic material and proteins, a substantial proportion of which is formed by different RNA species (Keerthikumar et al., 2016). Studies of PCa vesicle RNA indicate their potential as prognostic tools (Bryant et al., 2012; Li et al., 2014; Huang et al., 2015a; Foj et al., 2017; Rodriguez et al., 2017). For example, *TMPRSS2-ERG* and Prostate Cancer Antigen (*PCA-3*) mRNA, both PCa markers, have been found in EVs isolated from urine, demonstrating the composition of EVs could be used as diagnostic and prognostic indicators in biofluids (Rodriguez et al., 2017).

Our previous work demonstrated PCa cell line secreted EVs can enhance osteoblast viability, these PCa EV enhanced osteoblasts increase the growth of PCa cells when grown together in co-culture, demonstrating a potential mechanism of pre-metastatic bone cell modulation (Probert et al., 2019). The RNA cargo of PCa cell EVs is enriched in RNAs associated with cell surface signaling, cell–cell interaction, and protein translation, which we found can be transferred into osteoblasts, demonstrating for the first time the important contribution of the RNA element of the PCa EVs in modulating stromal cells within metastatic sites and providing a potential novel mechanism of mediating bone metastasis (Probert et al., 2019).

This study builds upon our previous *in vitro* studies by demonstrating that PCa EVs home to bone *in vivo*, and that over time exposure to PCa EVs mediates molecular changes within the bone that potentially represent the very earliest changes in the development of a pre-metastatic niche.

## 2 Methods

### 2.1 Cell lines and culture conditions

Cell lines PC3MLuc2a obtained from Xenogen (Perkin Elmer, UK) and hOB cells - hfOb1.19 obtained from ECACC (Culture Collections, UK) were cultured in Dulbecco’s modified medium (#D5796, Sigma, United Kingdom) supplemented with 10% fetal bovine serum (#B9433, Sigma, United Kingdom), 50 U mL−1 penicillin and 50 µg mL−1 streptomycin (#15140122, Gibco, UK). C42-4b (provided in collaboration with Dr Penny Ottewell, University of Sheffield) and PNT1A (were provided in collaboration with Professor Nigel Mongan, University of Nottingham) were cultured as described (Kashyap et al., 2013).

### 2.2 Isolation and characterization of EVs

Cells were seeding 1 × 10^6^ cells per 10 cm^2^ dish and incubated overnight. Following the overnight incubation, cells were washed in phosphate-buffered saline (PBS) and fresh media containing 10% exosome-free fetal bovine serum (#A2720801, Gibco, United Kingdom) applied. Cells were incubated for 48 h under standard conditions. After 48 h, the media was collected and subjected to centrifugation at 300 × g for 10 min, 2000× *g* for 10 min, 100,00× *g* for 30 min at room temperature. The resulting supernatant was concentrated to 0.5 mL using a Vivaspin‐20 (100 MWCO) device (Sigma Aldrich, United Kingdom). The resulting concentrated media was treated with Vibrant Dil Cell Labelling Solution (#V22885, Thermo-Fisher, UK) at 1µL/100 µL (v/v) and incubated at 37°C for 15 min. The labelled supernatant was then separated into fractions by gravity flow chromatography (qEV1-70, Izon Science, United Kingdom) following the manufacturers’ protocol.

EVs were characterized by electrophoresis and Brownian motion analysis using laser scattering microscopy (ZetaView, ParticleMetrix, Germany) and visualized by electron microscopy. Briefly, EV samples were fixed with 2% formaldehyde and 2 μL applied to glow discharged amorphous carbon transmission electron microscopy grids (200 mesh; Agar Scientific) and then placed onto a piece of parafilm in a humidity chamber for 30 min. The grids were then washed twice with 2 μL of ddH2O and then blotted dried. For negative staining, the TEM grids were placed onto a 10 μL droplet of 2% filtered uranyl acetate for 1-min, excess solution was blotted dry, and the grid allowed to air dry for 5 min. Transmission electron micrographs (Technai T12; FEI) were then taken of the grids containing the EV fractions at 4.8kx, 9.9kx, and 20.5kx magnifications (Supplementary Figure S1).

EVs were further characterized for EV cargo proteins. Total protein was determined using the Qubit Protein Assay Kit (ThermoFisher, United Kingdom) and the Qubit 3.0 instrument (ThermoFisher, United Kingdom) prior to Western blot. 20 μg of protein per EV sample and 10 μg per cell lysate were separated by SDS-PAGE on 10% polyacrylamide gels (BioRad, United Kingdom). Proteins were transferred to nitrocellulose membrane using the BioRad Transblot Turbo System. Membranes were blocked for 1 h in 5% (w/v) bovine serum albumin (BSA) in TBST. Primary antibodies were incubated for 18 h at 4°C at a 1:1,000 dilution in 3% (w/v) BSA in TBST as follows: CD9 (D801A), Alix (3A49), and GM130 (D6B1) (Cell Signaling, United States). Membranes were washed three times in TBST for 10 min, followed by a 60-min incubation with either HRP-conjugated anti-rabbit or anti-mouse secondary antibody (Cell Signaling, United States) at room temperature. Membranes were washed three times in TBST for 10 min, followed by incubation with Clarity ECL (BioRad, United Kingdom) and visualization using the BioRad GelDoc Go Imaging System (Supplementary Figure S1).

### 2.3 *In vivo* studies

The *in vivo* studies were carried out as described below. Briefly, CD1 NuNu male adult mice were injected via the tail vein with Dil labelled EVs in100 µL of PBS (equivalent to 60 µg total protein, median EV concentration of 2 × 10^7^), according to the schedule described below. Animals and organs were imaged at the appropriate time points using the IVIS Spectrum (Perkin Elmer, United Kingdom). Organs were snap frozen and stored at −80°C prior to downstream processing.

*In vivo* biodistribution experiments were performed to assess distribution and organ accumulation of the EVs. The experiments were conducted under the UK Home Office License number PPL P435A9CF8 with approval from the University of Nottingham AWERB. LASA good practice guidelines, FELASA working group on pain and distress guidelines and ARRIVE reporting guidelines were also followed.

10–12-week-old male CD-1 NuNu mice were purchased from Charles River UK. It was appropriate to only use males to model patient relevance. Mice were maintained in Individually Ventilated Cages (Tecniplast, United Kingdom) within a barriered unit, illuminated by fluorescent lights set to give a 12-h light-dark cycle (on 07.00, off 19.00), as recommended in the guidelines to the Home Office Animals (Scientific Procedures) Act 1986 (United Kingdom). The room was air-conditioned by a system designed to maintain an air temperature range of 21ºC ± 2°C and a humidity of 55% + 10%. Mice were housed in social groups, 4 per cage, during the study, with irradiated bedding and autoclaved nesting materials and environmental enrichment (Dates and, United Kingdom). Sterile irradiated 5V5R rodent diet (IPS Ltd., United Kingdom) and irradiated water (Baxter, United Kingdom) was offered *ad libitum*. The condition of the animals was monitored throughout the study by an experienced animal technician. After a week of acclimatization, the mice were randomly allocated by weight to the study groups of 2 mice per injection schedule. As this was a biodistribution study, no power calculation was required, minimum numbers used.

Prior to EV injection, the mice were optically imaged in the IVIS^®^ Spectrum imaging system, PerkinElmer (MA, United States), to obtain background control images. Prior to imaging, the mice were anaesthetized with an injectable anesthetic combination (Anaestemine [ketamine]/Sedastart [medetomadine], Animalcare Ltd. United Kingdom) before being placed in the imaging system. The mice were then injected with the EVs according to the schedule described below. After warming the mice in a thermostatically controlled heating box (Date sand United Kingdom), they were injected via the tail vein with Dil labelled EVs in100 µL of PBS (equivalent to 60 µg total protein). We selected the dosage based on previous optimization studies, attempting to tie together physiological relevance within the technical limitations of detection in our assays. In our hands, 60 µg of protein equated to a median EV count of 2 × 10^7^ per injection per mouse. Based on studies of plasma from human patients, circulating EVs have been reported as 2.92 × 10^10^ EV/mL plasma secreted from a variety of cellular sources (Johnsen et al., 2019). To date, the contribution of cancer EVs to the circulating population has not been defined due to variability in individuals and disease stage. However, our injection of EVs is within the physiological range for total amounts of circulating EVs and their accumulation remained detectable by IVIS.

Mice were imaged as above immediately post injection and then again prior to termination. No adverse effects were observed following the injections or for the duration of the study. At the appropriate study time point, the mice were terminally anesthetized, and blood was collected by intracardiac sampling and allowed to clot for serum collection. The mice were then killed by cervical dislocation and the organs harvested and imaged *ex vivo*. The organs excised and imaged are detailed in Table 1. Following imaging, organs and serum were snap frozen in liquid nitrogen. All imaging data was analyzed using the Living Image^®^ software (PerkinElmer, United Kingdom).

**TABLE 1:** Schedule of EV treatment.

Group	Treatment	Number of mice per group	Organs and tissues to collect
24 h of EV exposure	Vesicles injected on day 1 and terminated after 24 h	2 X adult CD1 nude male	Brain, heart, lung, liver, spleen, pancreas, kidney, lymph nodes, bladder, limb bones and urine sample
1 week of EV exposure	Vesicles injected twice a week for 1 week and animals terminated 24 h after final injection	2 X adult CD1 nude male	Brain, heart, lung, liver, spleen, pancreas, kidney, lymph nodes, bladder, limb bones and urine sample
2 weeks of EV exposure	Vesicles injected twice a week for 2 weeks and animals terminated 24 h after final injection	2 X adult CD1 nude male	Brain, heart, lung, liver, spleen, pancreas, kidney, lymph nodes, bladder, limb bones and urine sample
3 weeks of EV exposure	Vesicles injected twice a week for 3 weeks and animals terminated 24 h after final injection	2 X adult CD1 nude male	Brain, heart, lung, liver, spleen, pancreas, kidney, lymph nodes, bladder, limb bones and urine sample
4 weeks of EV exposure	Vesicles injected twice a week for 4 weeks and animals terminated 24 h after final injection	2 X adult CD1 nude male	Brain, heart, lung, liver, spleen, pancreas, kidney, lymph nodes, bladder, limb bones and urine sample
1 week PBS control injections	PBS injected twice a week for 1 week and animals terminated 24 h after final injection	2 X adult CD1 nude male	Brain, heart, lung, liver, spleen, pancreas, kidney, lymph nodes, bladder, limb bones and urine sample

### 2.4 *In Vitro* studies

Recipient cells (hOBs) were seeded into six-well plates at 2 × 10^5^ cells per well. Following an overnight incubation, the media was replaced with media containing 10% exosome-free fetal bovine serum (#A2720801, Gibco, United Kingdom) and cells were grown for 24 h prior to the application of isolated EVs.

### 2.5 RNA extraction and RNAseq

Femur, tibia, humerus, radius and ulnar limb bones were used after removing patella, fibula, and entirety of the feet. The bones were cleaned by cutting away all soft tissues, blood clots and blood vessels and other visibly contaminating material followed by aspiration with PBS. Visual observation was used to determine complete removal of any contaminating non-bone material. The tissue was cut into approximately 100 mg pieces and placed in the pre-packed FastPrep-24 2 mL microcentrifuge tubes (MP Biomedicals, United Kingdom). 0.6 mL of RNA protection reagent was added to each tube and the material homogenized at a speed of 4 m/s for 20 s in a Fast Prep Classic Homogenizer (MP Biomedicals, United Kingdom). If tissue was not completely homogenized, a subsequent 20 s pulse was performed. Homogenized samples were centrifuged at 12,000 × *g* (15 min, 4°C) to settle the protein precipitates and the supernatant was collected for RNA extraction. RNA was extracted using the Qiagen miRNAeasy kit according to the manufacturer’s instructions (Qiagen, United Kingdom).

For RNAseq, EV-treated mouse bone samples (bones from one fore and one hind leg per mouse) were sequenced by Novogene Co., LTD. (Cambridge, United Kingdom) using the Illumina NovaSeq platform to generate 150bp paired end reads. To determine if the potential molecular modulations of bone were detectable within the circulation, RNAseq data from circulating EVs isolated from whole blood from castration resistant PCa patients was analyzed [published (Huang et al., 2015b) accessed at GSE58410].

### 2.6 Data analysis

Data was analyzed using the Galaxy bioinformatics platform (Galaxy, 2022). RNAseq data of the EV-treated mouse bones, the sequence reads were trimmed with the TrimGalore wrapper for CutAdapt and mapped against the mm39 full genome and human hg19 using HISAT2 (Kim et al., 2015). 1.8%–2.29% of reads map to human. Cutoffs of <15% mapped reads to indicate sample contamination and <5% expected mapping when aligning to any similar species were used to concluded that human RNA sequences (contributed by EVs) fell within background levels and were without substantial contribution. Analysis was continued to determine differential expression based on mapping to the mm39 genome. Low-read filtering was applied to mapped data and differential expression determined using EdgeR on samples representing control and day 27 animals (Robinson et al., 2010). Due to *n* = 2 group numbers, stringent cutoffs for changes in genes were employed as a log2 fold change of ± 5 and FDR <0.1. Pathway enrichment analysis was performed using of QIAGEN IPA (QIAGEN Inc., https://digitalinsights.qiagen.com/IPA) and FunRich (Kramer et al., 2014; Pathan et al., 2015).

RNAseq data from circulating EVs isolated from whole blood from twenty-four castration resistant PCa patients was downloaded from GSE58410 (Huang et al., 2015b). The raw data was reanalyzed using the Galaxy bioinformatics platform. Sequence reads were trimmed with the TrimGalore wrapper for CutAdapt and mapped against the human hg19 using RNA STAR and FPKM used to calculate raw read count, FPM (fragments per million), and FPKM (fragments per million mapped reads per kilobase exon) for each gene (Wang et al., 2012; Dobin et al., 2013; Liao et al., 2014).

Additional statistical analyses were performed using GraphPad Prism version 10.0 for Windows (GraphPad Software, United States).

### 2.7 Immunohistochemistry and histological analysis

Upper and lower limb tissue was fixed in 4% paraformaldehyde for 2 h, dehydrated through an ethanol series, embedded into paraffin blocks, and sectioned at 7 μm. Immunohistochemistry was carried out on serial sections using a Leica Novolink Polymer Detection Kit (Leica, Germany) according to manufacturer’s protocols with primary antibodies diluted in 1:100 in fetal calf serum with an overnight incubation at 37°C. Rabbit monoclonals ab218237 [EPR21138] osteopontin (secreted phosphoprotein 1, SPP1, in humans), ab283654 [EPR23917-164] CD68, and ab93876 osteocalcin, rabbit monoclonals ab81289 [EP373Y] CD34 and ab290636 [EPR25122-122] SPARC, and mouse monoclonal ab88147 [3G3] Collagen I - BSA and azide free (Abcam, United Kingdom) were used to stain proteins of interest. In addition to immunohistochemistry, hematoxylin and eosin staining was conducted (2.5 min in hematoxylin, 15 s in 1% acetic industrial methylated spirits, 15 s in ammoniated water and 4 min in eosin). Picrosirius red staining was also performed to visualize collagen types I and III and undifferentiated collagens under polarized light (Polysciences, Inc., United States).

Microscopy was carried out to confirm positive staining (Leica CTR500 microscope, Leica Microsystems, Germany). Negative controls received no primary antibody and were incubated in fetal calf serum only, whilst skin, adipocytes, and muscle tissues were used as positive controls.

## 3 Results

### 3.1 Biodistribution of PCa EVs

**FIGURE 1:**
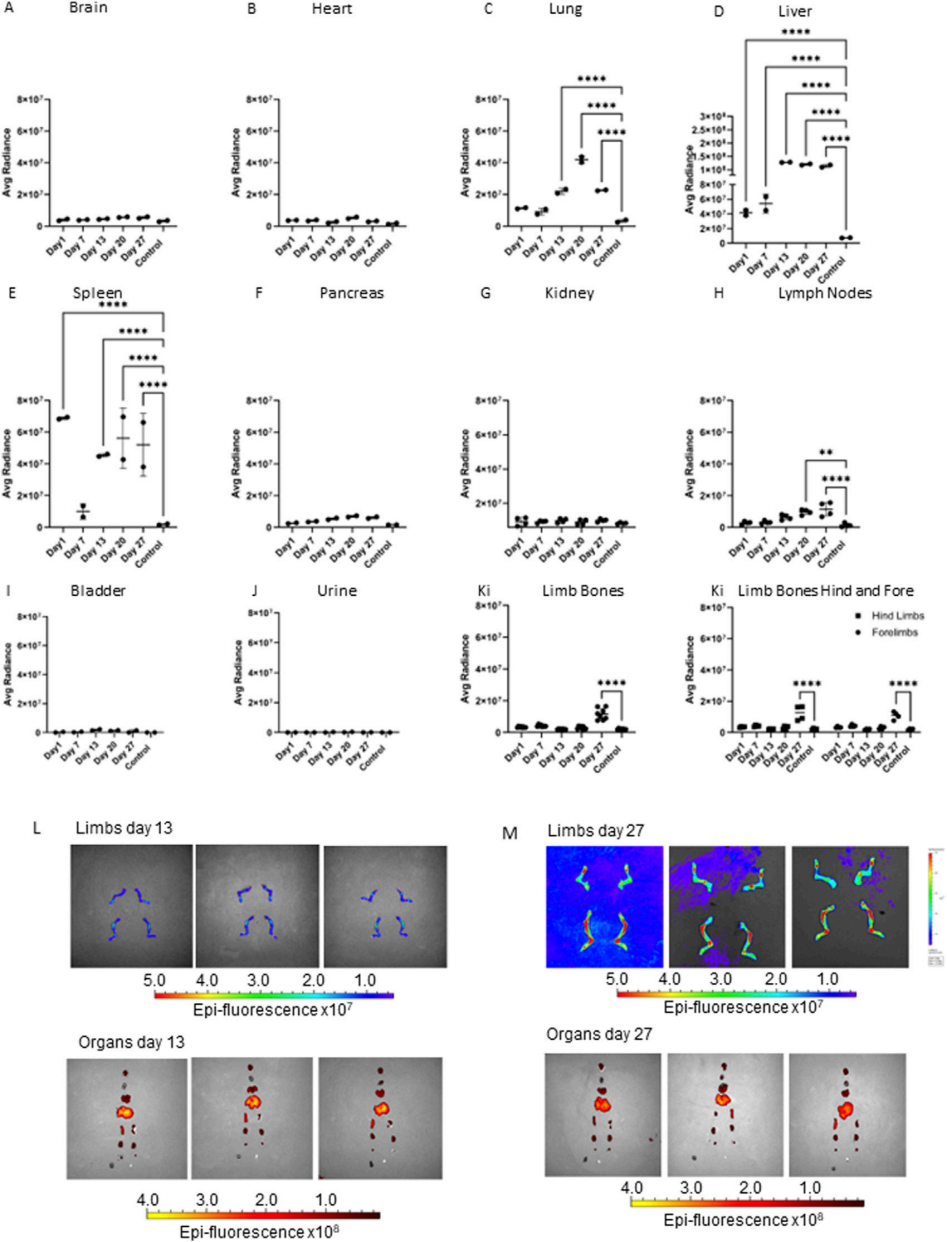
Accumulation of PC3MLuc EVs over 27 days in adult male CD1 NuNu mice. Accumulation of Dil labelled PC3MLuc EVs was determined via IVIS imaging at 1, 7-, 13-, 20- and 27-day timepoints in adult CD1 NuNu male mice exposed to EVs twice per week, compared to a vehicle only control. **(A)** brain, **(B)** heart, **(C)** lung, **(D)** liver, **(E)** spleen, **(F)** pancreas, **(G)** kidney, **(H)** lymph nodes, **(I)** bladder, **(J)** urine, and **(K)** limb bones. **(L, M)** IVIS imaging of organs and bones at days 13 **(L)** and 27 **(M)** in CD1 mice exposed to PC3MLuc EVs. Accumulation of EV signal can be seen in the joint area of the limbs, changing from low to high signal detection in the limbs of 27-day animals. Statistical significance was determined by 2-way ANOVA with Dunnett’s multiple comparison test, with single pooled variance. *<0.05, **<0.01, ***<0.001, ****<0.0001 (*n* = 2).

To determine the biodistribution of EVs and the organs they accumulate in overtime, Dil labelled EVs secreted from the PCa cell line PC3Mluc2a were injected via the tail vein of adult male CD1 NuNu mice. EVs were injected twice per week for up to 4-week and the accumulation of Dil signal in different organs was determined by IVIS imaging (Figures 1A–K). We identified a significant accumulation of signal in the liver and variable accumulation in the spleen from 24 h (Figures 1D, E). We also saw accumulations of signal in the lung after 2 weeks of EV exposure and in the lymph nodes and limb bones after three and 4 weeks of EV exposure. The later accumulation of signal in the lungs, lymph nodes and bone may represent a slower accumulation of the Dil signal in these organs compared to the liver and spleen or that the mechanism of accumulation in the non-elimination organs requires a specific homing process that required a longer period of EV exposure to manifest. We did not directly compare IV injection of EVs from a non-cancer cell line, as existing data from previous studies confirms non-cancer EVs do not commonly accumulate in bone. Grange et al., performed comparable studies using male CD1 nude mice injected with Dil labelled EVs isolated from non-cancer mesenchymal stem cells, no biodistribution to bone was detected using EVs in this model, whereas accumulation in the elimination organs was comparable to our data set (Grange et al., 2014). Furthermore, a meta-analysis of 38 EV biodistribution studies across other mouse models also confirms non-cancer EVs do not commonly accumulate in bone (Kang et al., 2021).

### 3.2 Molecular analysis of PCa EV-mediated changes in bone

**FIGURE 2:**
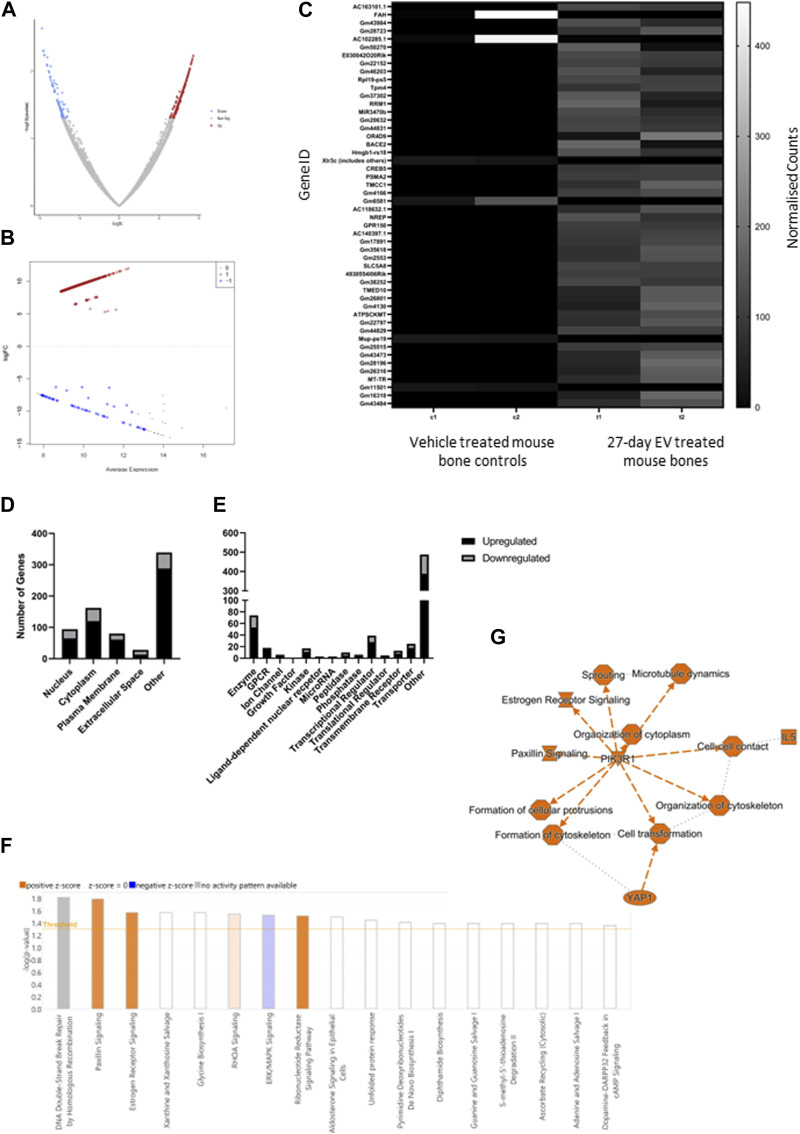
Summary of differential gene expression, gene function and pathway analysis in 27-day PC3MLuc EV treated bones. **(A)** Volcano plot to demonstrate cut-off selection for fold change and significance for up and downregulated genes. **(B)** Multidimensional scaling plot of analyzed gene set. **(C)** Heatmap of normalized count data for top fifty differentially expressed genes. **(D)** Cellular localization and **(E)** function of genes with significantly altered expression. **(F)** Pathway analysis identified an enrichment of genes showing altered expression in 25 different pathways, which predicted positive or inhibitory changes to those pathways predicted for 8 pathways (orange = positive Z or blue = negative Z scores). A summary of the pathway analysis predicts a pattern of changes summarized in **(G)**. Analysis generated by Qiagen IPA 2022.

The limb bones from animals treated with EVs for 4 weeks were analyzed by RNAseq. Over 97% of reads mapped to the mouse transcriptome indicating a minimum contribution from potential human PCa EV transcripts. The data was further filtered to remove low reads, using only genes exhibiting a log2 fold change of ± 5 and FDR <0.1 for the final differential expression analysis (Figures 2A, B). The top fifty differentially expressed genes are displayed in Figure 2C and showed predominantly increased expression. The cellular localization of dysregulated genes was predicted to be across all cellular compartments, with many proteins not confined to a single cellular space (Figure 2D). Of genes showing increased expression in bone following PCa EV exposure, 4.8% were linked with surface receptors and 2% with downstream kinase signaling, and 22% with enzymatic, transporter and gene regulatory activity (Figure 2E).

Pathway analysis identified gene enrichment that aligned with 17 pathways, of which the direction of change in gene expression was attributed a significant positive Z score for activation in four pathways (Paxillin signaling *p* = 0.0163, Estrogen Receptor signaling *p* = 0.0271, RHOA signaling *p* = 0.0287 and Ribonucleotide reductase signaling *p* = 0.0307) and a negative Z score for inhibitory activity in one pathway (ERK/MAPK signaling *p* = 0.0299) (Figure 2F). Taken together these predicted changes in pathway activation and inhibition suggested an increase in cell organization, cell-contact and dendritic protrusion formation (a factor that may be pertinent to osteocyte activity), together with changes in cell signaling networks for which phosphoinositide-3-kinase regulatory subunit alpha (*PIK3R1*) was identified as a central node (Figure 2G). Whilst not unique to bone, these pathways have been linked with bone homeostasis/remodeling, but these findings require further validation.

Based on the pathways determined, selected genes and regulators within these pathways were chosen to enable validation of the RNAseq data in a human *in vitro* model system. Expression of a subset of the genes identified (Table 2) were validated using human osteoblasts (hOB) grown *in vitro*. hOB cells were chosen as we had previously demonstrated PC3 EVs change the phenotype of hOB cells indicating they are a target recipient cell (Liu et al., 2016). hOB cells were exposed to EVs isolated from PC3MLuc2a and C42-4b prostate cancer cells and compared to treatment with EVs from PNT1A healthy prostate epithelial cells and EVs isolated from hOB cells.

**TABLE 2:** Summary of key regulatory genes identified by pathway analysis.

Gene	Regulation log2FC and FDR	Pathway involvement
*ESR1*	−12.81 (0.0473)	Estrogen receptor, ERK/MAPK, Ribonucleotide reductase
*SP7*	−12.489 (0.0314)	Osteoblasts in RA
*DKK1*	−10.969 (0.0000584)	Osteoblasts in RA, Osteoarthritis
*CSNK1A1*	−7.402 (0.0664)	Osteoblasts in RA
*SOS*	8.7 (0.00000878)	Paxillin, ERK/MAPK, EIF2, Integrin
*Talin*	9.4 (0.0000292)	Paxillin, ERK/MAPK, Integrin
*BCAR1*	9.173 (0.0000667)	Paxillin, ERK/MAPK, Integrin
*CREB*	9.806 (0.0000174)	Estrogen receptor, ERK/MAPK, Ribonucleotide reductase, Osteoarthritis
*ARHGEF6/7*	8.647 (0.0000298)	Paxillin, Integrin
*HIF1α*	9.155 (0.000108)	Ribonucleotide reductase, Osteoarthritis, Sirtuin
*CXCR2*	8.527 (0.0000447)	Osteoarthritis
*RRM1*	9.821 (0.0000174)	Ribonucleotide reductase
*GIT2*	9.821 (0.0000174)	Paxillin

Values in (), FDR.

PC3MLuc2a and C42-4b were chosen as both cell lines have bone metastatic capacity *in vivo*, although this propensity for bone differs as do the effects on bone due to the original derivation of the C42 line from an LNCaP (lymph node met cell line) subcutaneous xenograft. However, we were keen to see if these early EV-mediated changes were recapitulated by an alternative advanced PCa cell line. Despite published data indicating non-cancer EVs are unlikely to home to bone, we also used EVs from the non-cancer prostate epithelial cell line PNT1A. The use of the *in vitro* system enabled the forced interaction between PNT1A EVs and bone cells, that otherwise may not occur *in vivo*, determining if the EV-mediated changes were prostate cancer specific. Finally, EVs derived from hOB cells were used as a control for the addition of EV material, as this is the addition of the cells own EVs at the same concentration as the other EV types, the use of this control informed us if the changes observed were simply the result of the addition of a higher concentration of EVs rather than being specific to the origin of the EVs.

**FIGURE 3:**
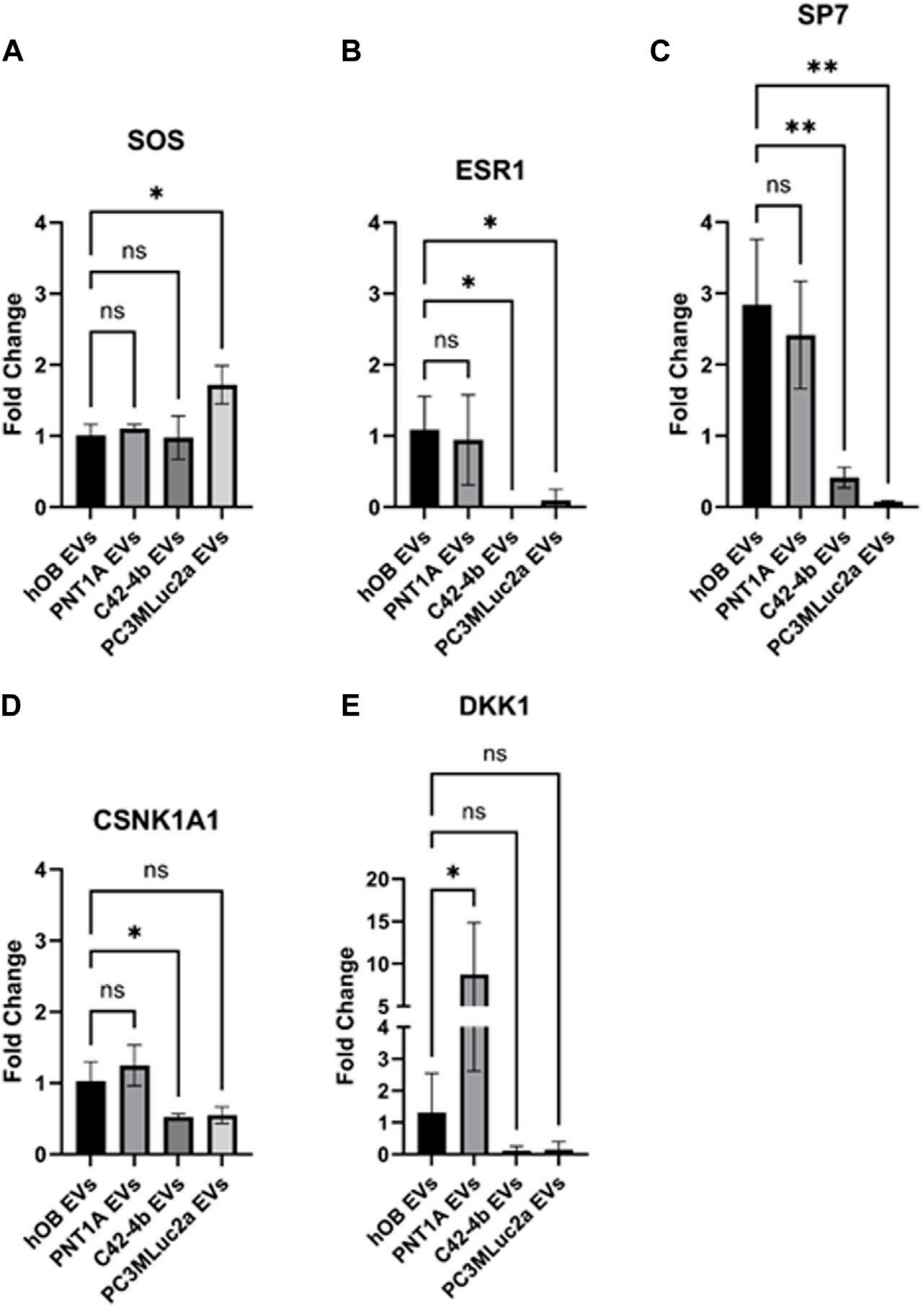
Quantitative PCR validation of altered gene expression following exposure to PC3MLuc2a-EVs using an *in vitro* osteoblast model system. Human osteoblasts (hOB) were exposed to EVs isolated from PC3MLuc2a, C42-4b prostate cancer cells or PNT1A healthy prostate epithelial cells of from other hOB cells grown in culture. The expression of **(A)** SOS, **(B)** ESR1, **(C)** SP7, **(D)** CSNK1A1 and **(E)** DKK1 were confirmed by qPCR. *n* = 3 biological replicates. Significance determined by one-way ANOVA with Dunnett correction. *p < 0.05, **p < 0.01.

The expression of *SOS*, *ESR1*, *SP7*, *CSNK1A1*, and *DKK1* were confirmed by qPCR. For *SOS*, we confirmed a significant increase in expression following hOB exposure to PC3MLuc2a EVs (*p* = 0.01) (Figure 3A). *ESR1* and *SP7* were significantly downregulated following hOB exposure to PC3MLuc2a EVs (*p* = 0.04 and *p* = 0.0019 respectively) (Figures 3B, C), and whilst not reaching significance *CSNK1A1* and *DKK1* were also downregulated (Figures 3D, E). Exposure of hOBs to C42-4b EVs showed similar changes, except for SOS which showed no change in expression (Figures 3A–E). PNT1A EVs did not cause an increase in SOS expression or a significant reduction in expression of *ESR1*, *SP7*, *CSNK1A1* or *DKK1*. However, we did observe that PNT1A EVs had the opposite effect for *DKK1*, causing a significant increase in *DKK1* expression (*p* = 0.048) (Figure 3E).

**FIGURE 4:**
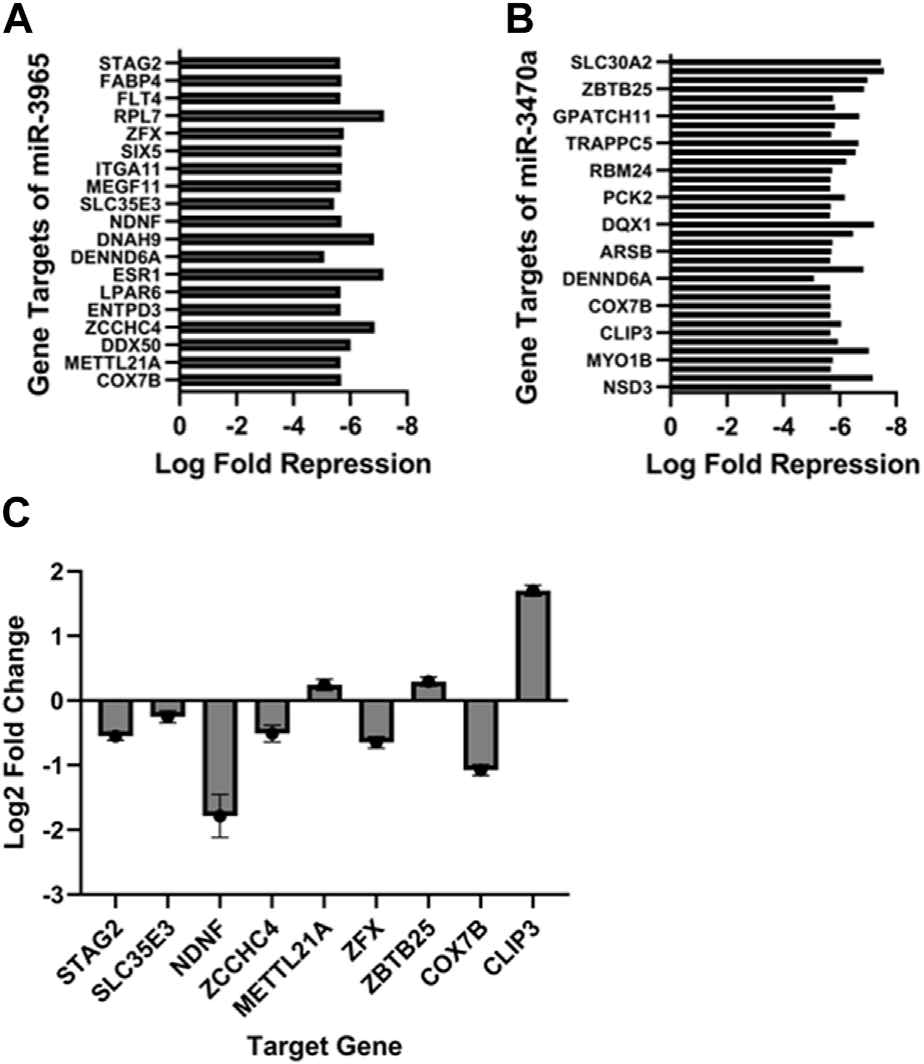
Upregulation of miR-3965 and miR-3470a with corresponding target suppression in 27-day PC3MLuc2a-EV treated mouse bones. miR-3965 and miR3470a (analogous to human miR21-5p and miR1285-5p) were found to have an increased expression in 27-day PC3MLuc2a-EV treated mouse bones. Target genes of both microRNAs were found to be simultaneously downregulated in the same EV-treated mouse bones **(A)** targets of miR-3965 **(B)** targets of miR3470a. **(C)** Gene targets which are the same in both mice and humans (STAG2, SLC35E3, NDNF, ZCCHC4, METTL21A, ZFX, ZBTB25, COX7B, and CLIP3) for these two miRNAs were examined *in vitro* treatment via the treatment of human osteoblasts (hOBs) with EVs isolated from PC3MLuc2a cells compared to EVs from control PNT1A cells. All results were repeated in triplicate and significant at *p* < 0.0001, except for SLC35EB *p* = 0.016 as determined by Wald test for significance.

In addition to changes in mRNA expression, two microRNAs were also identified as showing significantly increased expression in PCa EV treated bone. *miR3965* and *miR3470a*, which have sequence similarity to human *miR21-5p* and *miR1285-5p,* respectively. The downstream targets of both miRNAs were identified in the bone RNAseq dataset, demonstrating the activity of these enhanced miRNAs to modulate key regulators of bone remodeling (Figures 4A, B). To validate these findings, we identified a selection of genes which are targeted by these miRNAs and their human counterparts in both mice and humans (STAG2, SLC35E3, NDNF, ZCCHC4, METTL21A, ZFX, ZBTB25, COX7B, and CLIP3) and determined if the *in vitro* treatment of human osteoblasts (hOBs) with EVs isolated from PC3MLuc2a cells compared to EVs from control PNT1A cells resulted in the same repression of these targets. Except for METTL21A and CLIP3, all targets showed significant repression (*p* < 0.0001) (Figure 4C).

**FIGURE 5:**
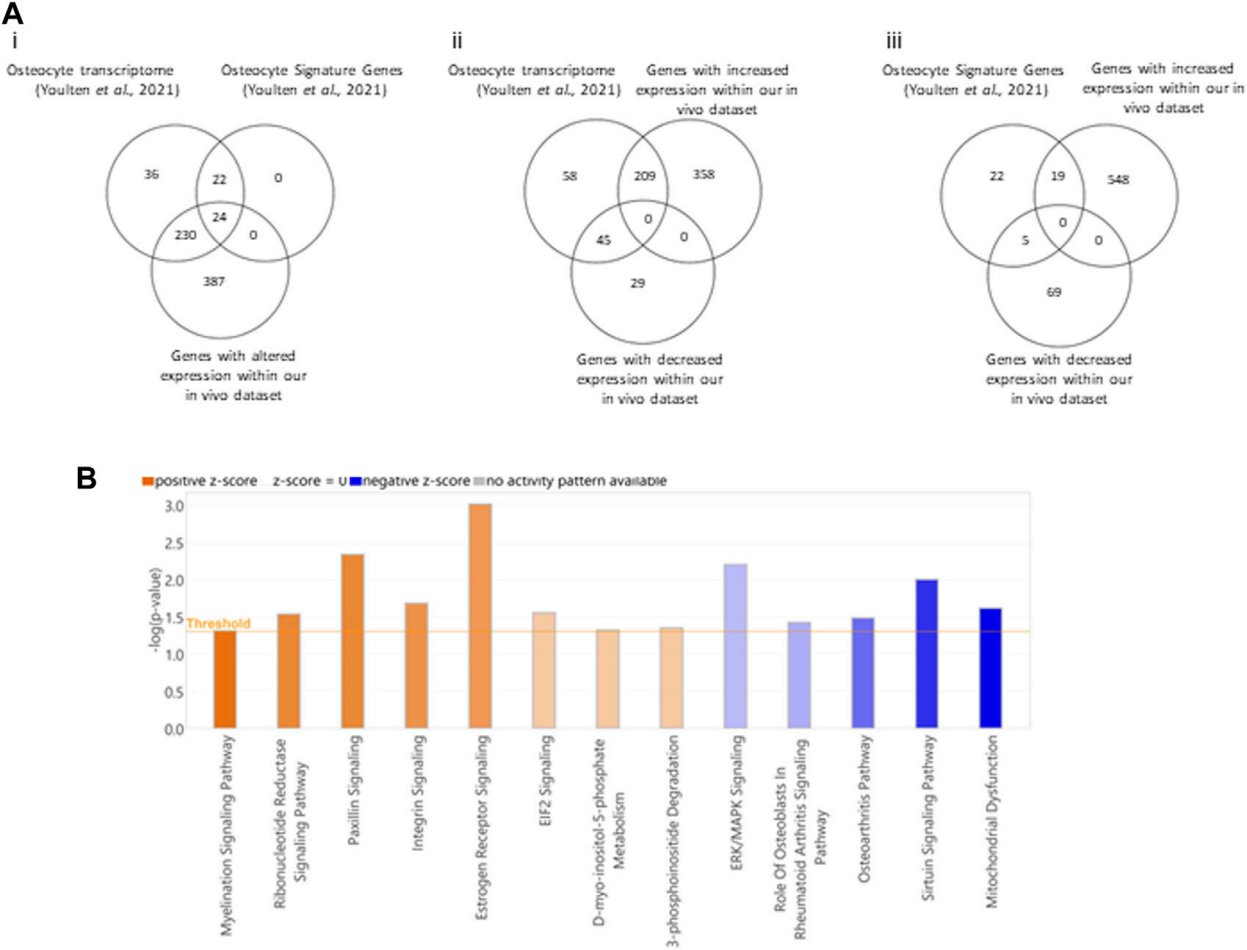
Comparison of transcript profiles between 27-day PC3MLuc EV treated bones and isolated unmodified mouse osteocytes. [**(A)**–i] comparison of both transcript profiles shows 39.6% of genes dysregulated in EV-exposed mouse bones (Our *in vivo* Dataset) were also found in the transcriptome of mouse osteocytes (Osteocyte Transcriptome, Youlten et al., 2021). When comparing the gene signature identified as unique to mouse osteocytes (Osteocyte Signature Genes, Youlten et al., 2021), the overlap in dysregulated gene expression increases to 52.2%. (ii) Comparison between the Osteocyte Transcriptome and differentially expressed genes in our EV-exposed mouse bones separated as up or downregulated genes from our *in vivo* dataset. (iii) Comparison between Osteocyte Signature Genes and genes from our *in vivo* dataset of EV-exposed mouse bones separated as up or downregulated genes. **(B)**. Pathway analysis of 254 genes that overlap between Osteocyte Transcriptome and our *in vivo* dataset of EV-exposed mouse bones (orange = positive Z or blue = negative Z scores). Pathway Analysis generated by Qiagen IPA 2022.

Given the genes with altered expression in bone following PCa EV exposure were enriched in pathways linked with cell-cell contact and protrusion formation, we wanted to confirm if there was evidence to support osteocytes being a contributor to the molecular changes being induced by the PCa EVs. Osteocyte processes contact walls of canaliculi at discrete points within the mineralized matrix and relay cell signaling in response to bone stress, making them central in remodeling processes (McNamara et al., 2009). Youlten et al. (2021) characterized the gene expression of mouse osteocytes and defined a unique gene signature using RNAseq data (Youlten et al., 2021). We determined whether genes known to be expressed in enriched mouse osteocyte populations featured within the aberrant gene expression profile identified in our PCa EV treated mouse bones. 39.6% of the dysregulated genes in the PCa EV treated bones aligned with the transcriptome of mouse osteocytes (Figure 5Ai–ii). When comparing with the unique gene signature identified by Youltan et al., 52.2% of the genes overlap with our dataset from the PCa EV treated bone (Figure 5Aiii). These findings suggested osteocytes may be a major contributor to the molecular changes mediated by PCa EVs.

To identify what contributions may be made by this subset of genes which potentially originate in the osteocyte population, we performed pathway enrichment analysis on this subset of genes only. We identified enrichment of genes in thirteen pathways for which the associated Z-score suggests activation or inhibition (Figure 5B). Positive Z-scores were attributed to Myelination signaling (*p* = 0.0487), ribonucleotide reductase signaling (*p* = 0.0292), Paxillin signaling (*p* = 0.00462), Integrin signaling (*p* = 0.0209), Estrogen receptor signaling (*p* = 0.000972), EIF2 signaling (*p* = 0.028), D-myo-inositol-phosphate metabolism (*p* = 0.048) and 3-phosphoinositide degradation (*p* = 0.0446). Negative Z-scores for ERK/MAPK signaling (*p* = 0.00632), Osteoblasts in rheumatoid arthritis signaling (*p* = 0.0378), Osteoarthritis pathways (*p* = 0.0329), Sirtuin signaling (*p* = 0.01) and mitochondrial dysfunction (*p* = 0.0243).

### 3.3 Histological analysis of PCa EV-mediated changes in bone

**FIGURE 6:**
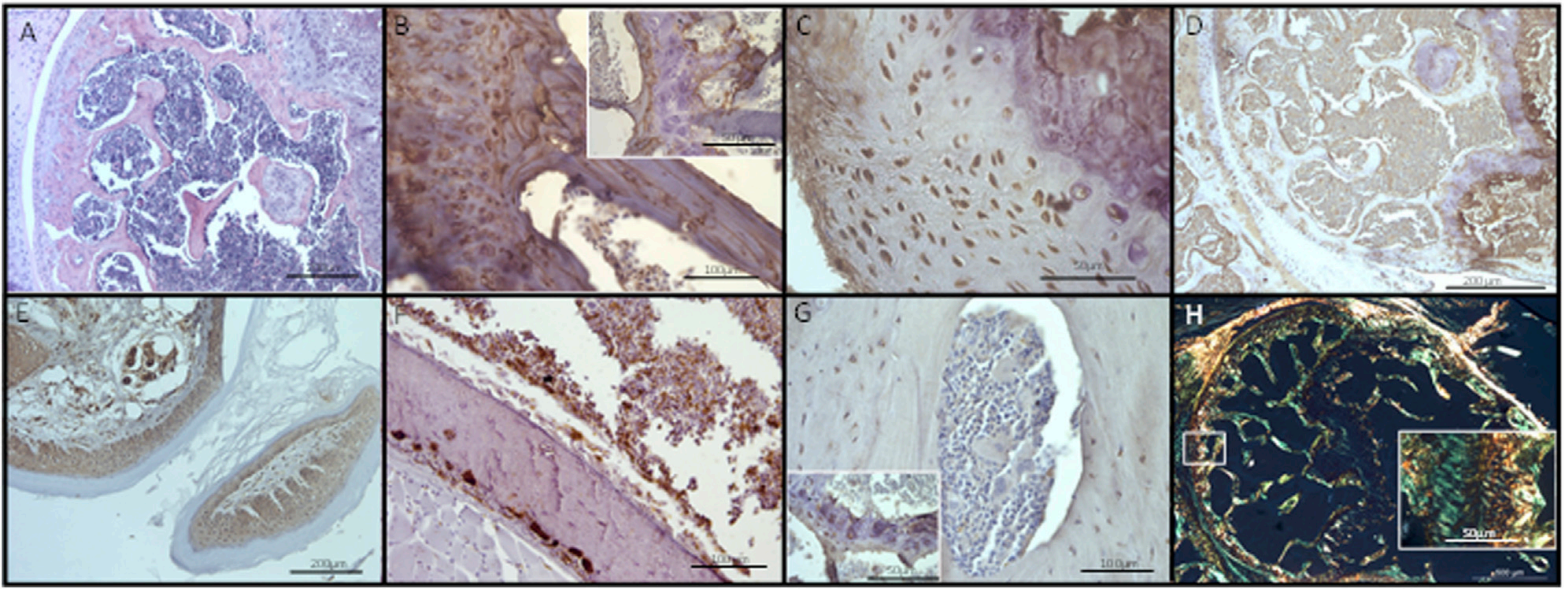
Histological analysis of EV treated bone sections. No changes in bone architecture were observed following visualization using **(A)** H&E staining, or immunohistochemistry using markers for **(B)** osteopontin, **(C)** osteocalcin, **(D)** SPARC, **(E)** CD34, **(F)**, CD68, **(G)** collagen I, or **(H)** the special histological stain picrosirius red (polarized light). Type I collagen stained yellow; type III collagen stained green. Scale bars: A, D, E = 200 µm, B, F, G = 100 µm, C, insets for B, G, H = 50 µm, H = 500 µm. Control images can be found in Supplementary Figure S2.

Given the evidence of molecular changes, we sought to ascertain if these changes had yet translated into histological alterations within the bone. Qualitative analysis by H&E and with markers osteopontin, CD68, osteocalcin, CD34, collagen I and picrosirius red did not identify any changes in the bone architecture at the histological level (Figure 6; Supplementary Figure S2). Bone, bone marrow, and hypertrophic chondrocyte immunohistochemistry staining for osteopontin (secreted phosphoprotein 1, SPP1, in humans), osteocalcin and SPARC (osteonectin), no staining was observed in the non-hypertrophic chondrocytes (Figure 6; Supplementary Figure S2). CD34 and CD68 were present in bone and cartilage tissue, whereas collagen I was not expressed in the cartilage but was expressed in bone tissues, as confirmed by IHC and picrosirius red staining, within the EV treated mice (Figure 6; Supplementary Figure S2). These results indicated normal histological development of both hypertrophic and non-hypertrophic chondrocytes, and bone tissues, within the EV treated mice. We did not observe any gross changes in osteocyte peri-lacunocanalicular remodeling. However, due to limited material, specific studies to explore this further were not conducted and we could not conclusively rule out early changes in the lacunocanalicular network. These findings suggested the molecular changes identified are early changes occurring prior to any substantial bone remodeling activity. Therefore, if these molecular changes are creating a pre-metastatic niche, screening for these early changes in patients may provide a window of opportunity to block the progression of the metastatic cascade.

### 3.4 EV-mediated molecular changes in bone as early indicators of potential bone-metastasis

**FIGURE 7:**
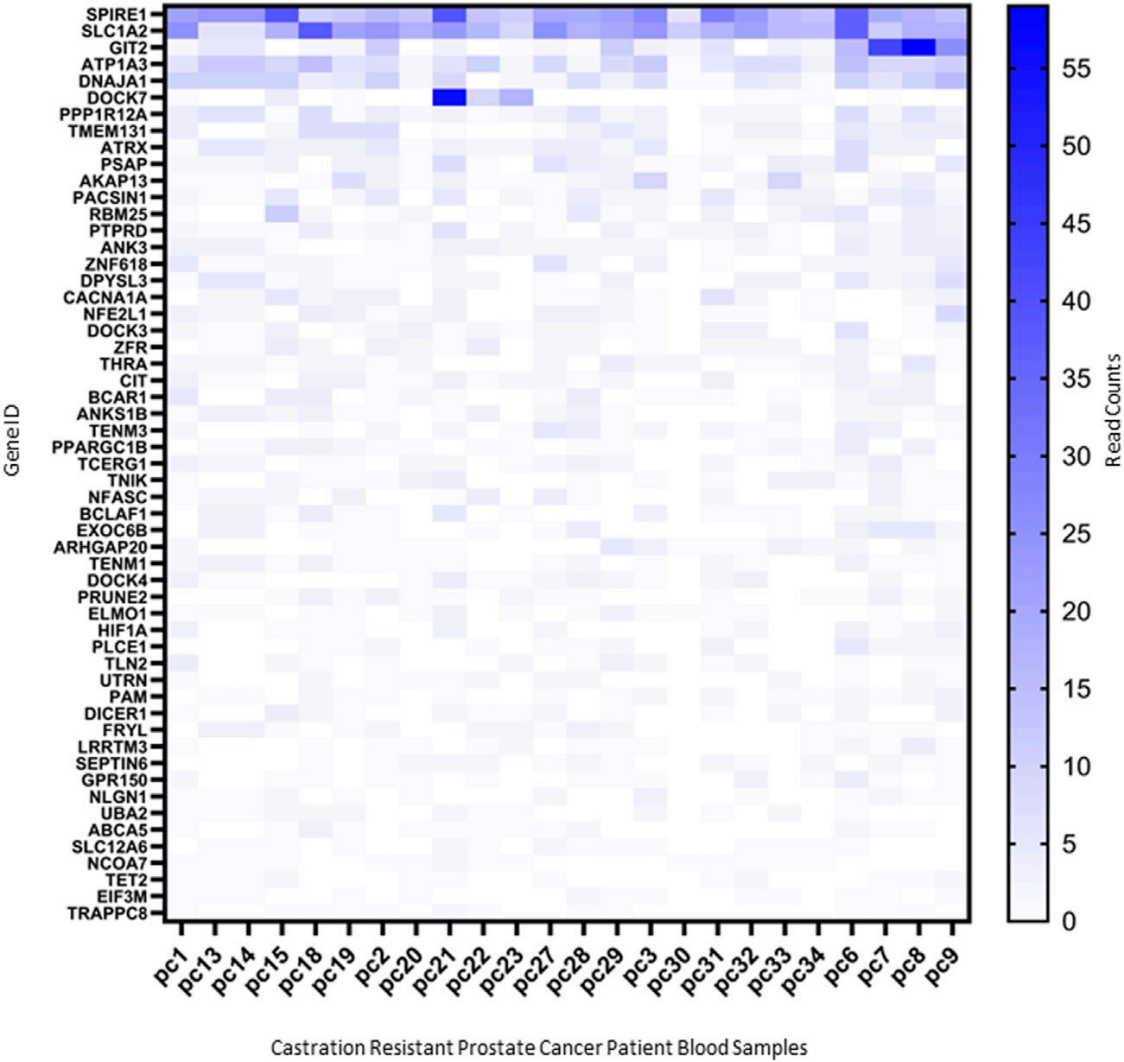
Markers identified in 27-day PC3MLuc2a-EV treated mouse bones detected within circulating EVs from castration-resistant patients. Molecular changes identified in 27-day PC3MLuc2a-EV treated mouse bones, were found to be detectable in circulating EVs isolated from the whole blood of patients with castration resistant PCa. 55 RNAs were detectable in RNAseq data produced from these samples based on read counts. Data accessed from GSE58410.

To determine the potential for using the molecular changes identified as early markers to identify patients at risk of bone metastasis, we sought to determine whether any of these changes were detectable within the circulation of patients with castration resistant PCa. RNAseq of circulating EVs isolated from twenty-four patients with castration resistant PCa, expressed 55 RNAs and one miRNA previously found to be increased in mouse bone exposed to PCa EVs for 27 days (Figure 7). Whilst these molecular changes are not unique to the creation of a bone-metastatic niche, the existence within the circulation warrants further investigation as to their utility as early indicators of the development of a pre-metastatic niche, either via sampling blood or bone directly.

## 4 Discussion

*In vivo* studies to determine EV biodistribution identified EV accumulation in the liver and spleen from 24 h post EV injection. We also saw accumulations of signal in the lung after 2 weeks of EV exposure and in the lymph nodes and limb bones after three and 4 weeks of EV exposure, respectively. Metanalysis of nineteen published studies using IV injection of EVs identified that at 24 h post EV exposure, high-to-moderate accumulation of EVs in the liver, spleen, lungs, and kidney was a common finding in 80% of the studies. Whilst brain, heart, bone, bladder, and pancreas were observed to have low to no accumulation of EVs. Unlike the accumulation seen in the liver, spleen, lungs and kidneys, accumulation in the brain, heart, bone, bladder, and pancreas was dependent upon the original source of the EVs and animal model (Kang et al., 2021). Our data shows a similar high-to-moderate accumulation of EVs in lung, liver, and spleen. When compared with the data analyzed in Kang et al. (2021), this suggests that the accumulation in these sites is likely a result of the IV administration of EVs and routes of nanoparticle elimination (Lai et al., 2014; Gangadaran et al., 2017). Whereas the increase in lymph nodes and limb bones at the later stages of the study, might more likely be linked with the PCa origin of the EVs and a specific mechanism of homing to these tissues (Kang et al., 2021). The lack of significant EV accumulation in the brain, heart, pancreas, and bladder provides further evidence to support the significant changes in lymph nodes and limb bones being likely due to the EVs originating from PC3MLuc2a prostate cancer cells and specific homing mechanisms that make the lymph nodes and bone target organs for prostate cancer metastasis *in vivo* (Jenkins et al., 2003).

Molecular analysis of the long bones of EV treated animals by RNAseq identified changes in pathways critical to bone homeostasis and remodeling. Of note, activation of Paxillin, Estrogen-receptor signaling, Ras homolog family member A (*RhoA*) signaling, ribonucleotide reductase signaling and a reduction in Extracellular signal-regulated kinases/Mitogen-activated protein kinases (ERK/MAPK) signaling. These signaling pathways function in various non-bone cell populations, as well as having roles in bone cell regulation. Paxillin acts to regulate focal adhesion formation and interacts with other signaling pathways, such as Rho-GTPase signaling, to mediate cytoskeletal organization and cell migration in response to physical or biochemical stimuli the functions of which can regulate bone remodeling activity through modulation of osteoclast and osteoblast activity (Deakin et al., 2012; Zou et al., 2012; Deakin and Turner, 2014; Tang et al., 2018). Furthermore, RhoA also regulates age-associated bone loss through destabilization of *β*-catenin via activation of Janus kinase (Jak) phosphorylation of the Tyr216 residue of glycogen synthase kinase-3β (*Gsk3β*) (Shi et al., 2021). Our data indicate downregulation of Estrogen Receptor 1 (*ESR1*) expression, which would be predicted to reduce the antagonistic effects of *ESR1* on *ESR2*, promoting an increase in *ESR2*-mediated mechanisms of osteoclast suppression and osteoblast activation whilst a reduction in ERK/MAPK signaling in osteocytes and mature osteoblasts is linked with increased bone formation through dysregulated osteoblast maturation and osteocyte apoptosis (Kameda et al., 1997; Garcia et al., 2013; Nelson et al., 2014; Martin et al., 2015; Kim et al., 2022). Taken together, the enrichment of the pathways predicted by a significant Z score indicates the potential for early changes associated with bone remodeling. However, it must be noted that these pathways have a variety of distinct functions, including the regulation of the immune response, which is lacking in this model, going beyond regulation of osteoblasts and osteoclasts alone.

To validate these findings, we used an *in vitro* assay to treat human osteoblasts in culture with EVs produced by PC3MLuc2a and C42-4b prostate cancer cells, comparing to EVs produced by non-cancer prostate epithelial cells PNT1A and EVs produced by osteoblasts. Changes in expression of genes enriched within the identified pathways were analyzed and we found similar changes in expression for *SOS*, *ESR1*, *SP7*, *CSNK1A1* and *DKK1* as seen in the *in vivo* EV-treated mouse bones, except for EVs isolated from C42-4b cells, which did not elicit an effect on *SOS* expression. As C42-4b has been shown to be bone metastatic *in vivo*, a similar effect of C42-4b EVs supports the hypothesis that EVs contribute to pre-metastatic niche formation in bone. The origin of the C42-4b cell line being derived from the lymphatic metastatic line LNCaP, and its different propensity for bone *in vivo* and different effects of the tumor cells on bone (generating osteoblastic rather than osteolytic lesions), may give rise to the subtle difference seen between C42-4b and PC3MLuc2a EVs—this requires further investigation. We also used EVs isolated from healthy prostate epithelial cell line PNT1A. Our *in vitro* data shows treatment with EVs isolated from PNT1A cells produced no significant effect on the expression of the target genes within the osteoblasts, except for *DKK1*. Evidence from published *in vivo* studies suggest EVs from non-cancer cell lines are not commonly biodistributed to bone and in a healthy individual, it seems unlikely that EVs produced from the non-cancerous prostate would reach the bone in significant numbers, therefore the effects of PNT1A EVs *in vitro* would not necessarily be considered normal. However, the role of *DKK1* requires further investigation as recent studies by Eyre *et al.*, identified a role for *IL1β* expression within bone promoting cancer colony formation via activation of *NFKβ*, *CREB* signaling and *WNT* ligand secretion. Expression of *DKK1* can reverse this effect and patients with breast cancer bone metastases that exhibit high levels of *DKK1* have better outcomes. Therefore, whilst we did not find elevated levels of IL1B in our samples, the effect of prostate cancer EVs to lower *DKK1* expression may lead to priming of the bone to tackle increase in *IL1β* expression and *WNT* signaling after arrival of the tumor cells, whereas potentially healthy prostate tissue may have a beneficial influence on bone health through elevating *DKK1* levels (Eyre et al., 2019).

These data validate the *in vivo* findings and support the hypothesis that the prostate cancer origin of the EVs is required to mediate the changes in gene expression we observed both *in vitro* and *in vivo*. This is further confirmed by the use of the same concentration of EVs isolated from osteoblasts, showing that a bolus of EVs alone does not reproduce the effect. These data together strongly indicated the molecular changes observed are the result of the prostate cancer origin of the EVs.

Two miRNAs, *miR3965* and *miR3470a* which have sequence similarity to human *miR21-5p* and *miR1285-5p* respectively, were identified as being increased in expression. *miR1285-5p* and *miR21-5p* have both been linked with regulating bone metabolism and homeostasis through regulating genes involved in differentiation and function of osteoblasts and osteoclasts respectively (Hu et al., 2017; Pao et al., 2021). In addition, *miR21-5p* has been linked with PCa progression, specifically the development of bone metastasis by mediating the resistance of bone cells to apoptosis (Hu et al., 2017). The decreased expression of such genes by either or both miRNAs were confirmed in the PCa-treated mouse bones. Treatment of human osteoblasts *in vitro* with EVs isolated from PC3MLuc2a compared to non-cancer PNT1A EVs also confirmed repression of targets of these miRNAs. The increased abundance of these miRNAs and corresponding suppression of their target genes following exposure to PCa EVs, suggests a key role for these miRNAs in mediating the effects of PCa EV mediated communication that warrants further investigation.

Further analysis also identified a correlation between genes dysregulated in our dataset and the gene signature of osteocytes. Whilst bone remodeling is predominantly associated with osteoblasts and osteoclasts, the osteocytes have an established role in orchestrating bone homeostasis and remodeling through stimuli sensing (Youlten et al., 2021). Substantial overlap in the gene signature for osteocytes was present within our bone RNAseq dataset, suggesting the potential contribution of this cell type in response to PCa EVs, these genes are enriched in pathways with reported roles in osteocyte function. For example, a relationship between osteocytes and the ECM via the Paxillin pathway, with the intracellular mediator of alpha and beta integrins, Talin, showing increased expression. Alongside, activation of actin regulation for filopodia and lamellipodia dendritic-like protrusions of the osteocytes used for sensing, Paxillin and *β*1 and *β*3 integrin signaling have been shown to be required for osteocyte mechanotransduction response to fluid shear stress. Dysregulation of both *β*1 and *β*3 integrin signaling has been linked with bone loss and disease states, including bone metastasis (Zhang et al., 2018; Geoghegan et al., 2019). Collectively, the pathways analyzed show loss of *ESR1* as a major contributory factor to changes in several signaling networks. Downregulation of *ESR1* in osteocytes has been shown to regulate trabecular bone formation and thereby trabecular bone volume in male mice via these pathways, with subsequent bone loss being linked with disseminated prostate tumor cell growth (Windahl et al., 2013; Ottewell et al., 2014). The general downregulation of ERK/MAPK signaling seen within the dataset, whilst surprising, may represent priming/alteration of the osteocytes and other bone cells rather than activation generally seen with a sheer stress response. Impaired ERK/MAPK signaling is known to disrupt the formation of osteocyte-lacunar-canalicular system, via loss of Dmp1 expression and osteocyte differentiation, resulting in impaired bone homeostasis (Kyono et al., 2012). The remaining pathway analysis data shows changes in transcriptional activity, through changes in factors cAMP response element-binding protein (*CREB*), *ESR1* and hypoxia-inducible factor-1 alpha (*HIF1α*). These predicted pathways require further validation but demonstrate a growing body of evidence to support the role of osteocytes in pre-metastatic niche formation. The role of osteocytes during prostate cancer bone metastasis has recently been established (reviewed in Maroni and Bendinelli (2020)).

We sought to determine if the molecular changes observed had already translated into clear changes in the bone architecture at the histological level. In keeping with our hypothesis that PCa EVs mediate molecular changes representing the very earliest changes in the bone, priming the bone for subsequent tumor cell arrival, we did not determine any qualitative histological changes in the bone architecture. Recent studies have shown that osteocytes can directly alter their local bone architecture through peri-lacunocanalicular remodeling, a process highly regulated by TGF- *β* signaling (Schurman et al., 2021). Disruption of this osteocyte network has been observed as the pre-metastatic niche develops (Hemmatian et al., 2021). We did not observe changes to the network within our histological analysis or any changes to TGF-β signaling, however, we cannot conclusively rule out more subtle alterations. Further studies, including microCT would help to uncover if PCa EVs are able to induce more subtle changes in lacunocanalicular remodeling, further supporting the role of osteocytes in an EV-mediate pre-metastatic niche. Further studies are also needed to quantify any specific changes in specific bone cell types, e.g., activation of osteocytes. However, our current data supports the hypothesis that detecting these early molecular changes in patients may provide a window of opportunity to halt the metastatic cascade, prior to substantial bone remodeling, preventing tumor cell colonization.

Finally, we determined if evidence of the molecular changes occurring in bone could be detected in patients. Using existing datasets of circulating RNAs (within EVs) from patients with castration resistant PCa, we determined the detectable presence of 55 RNAs and *miR21-5p*. Whilst many of these RNAs may be circulating from a variety of sources and may also be present within non-cancer populations, combinations of these molecules specifically linked with bone remodeling may provide a potential means of monitoring pre-metastatic niche development. For example, SPIRE1 was found to be one of the most abundant of the RNAs analyzed and has known roles in immunomodulation of bone marrow mesenchymal stem cells (Shao et al., 2023). Further research into this area may provide the potential to identify patients at risk of bone metastasis at the earliest stages.

### 4.1 Limitations

This study has used an immunodeficient mouse model to enable the use of human PCa cell lines. A major limitation within the field is generating reliable bone metastases *in vivo*, which only occur with the use of selected human prostate cancer cell lines. However, our data has uncovered the alteration of pathways that are involved not only in bone-remodeling, but also potentially in the immune response if it were present. Whilst the use of immunocompromised mice does not negate the findings of the study, an awareness that a major element of the metastatic process is absent within these animals needs to be kept in mind. Future studies need to account for how the immune system may be altered by PCa EVs, to ensure key signals have not been missed and to understand the growing role of immune-based therapy in PCa. Further studies are also needed to evidence the mechanistic insights uncovered by the study, particularly validating the role of osteocytes and contributions of other bone cells in response to PCa EVs.

## 5 Conclusion

We have determined that PCa EVs can home to both bone and lymph nodes within immunocompromised mouse models. Furthermore, the accumulation of EVs within the bone leads to molecular changes which may be attributable to early stages of disrupted bone homeostasis. Further investigation is required to confirm these changes and the possible role for osteocytes, ascertaining how early osteocyte changes may fit with established evidence for their role during clinically detectable bone metastasis. These findings require further validation to determine the underpinning mechanisms and temporal changes but contribute to a growing body of evidence supporting a mechanistic role for PCa EVs in PCa bone metastasis. Given the advancements in therapeutic options for patients, including chemotherapeutic agents, new-generation hormone therapies, radium 223 and, more recently, radioligand therapies. Understanding how the pre-metastatic niche in bone is formed and early markers of this process would enable timely therapeutic interventions in patients at risk. Furthermore, identifying patients at risk of bone metastasis at the earliest possible stage enables the directed management of their bone health and prevention of treatment-induced bone loss.

## Data Availability

The datasets presented in this study can be found in online repository NCBI SRA, accession numbers PRJNA252516 and PRJNA1073431.
