# Supplementary figures and images for: Modulation of the pre-metastatic bone niche: molecular changes mediated by bone-homing prostate cancer extracellular vesicles
Source: Front Cell Dev Biol. 2024 Feb 22;12:1354606. doi: 10.3389/fcell.2024.1354606 (PMC10919403; doi:10.3389/fcell.2024.1354606)

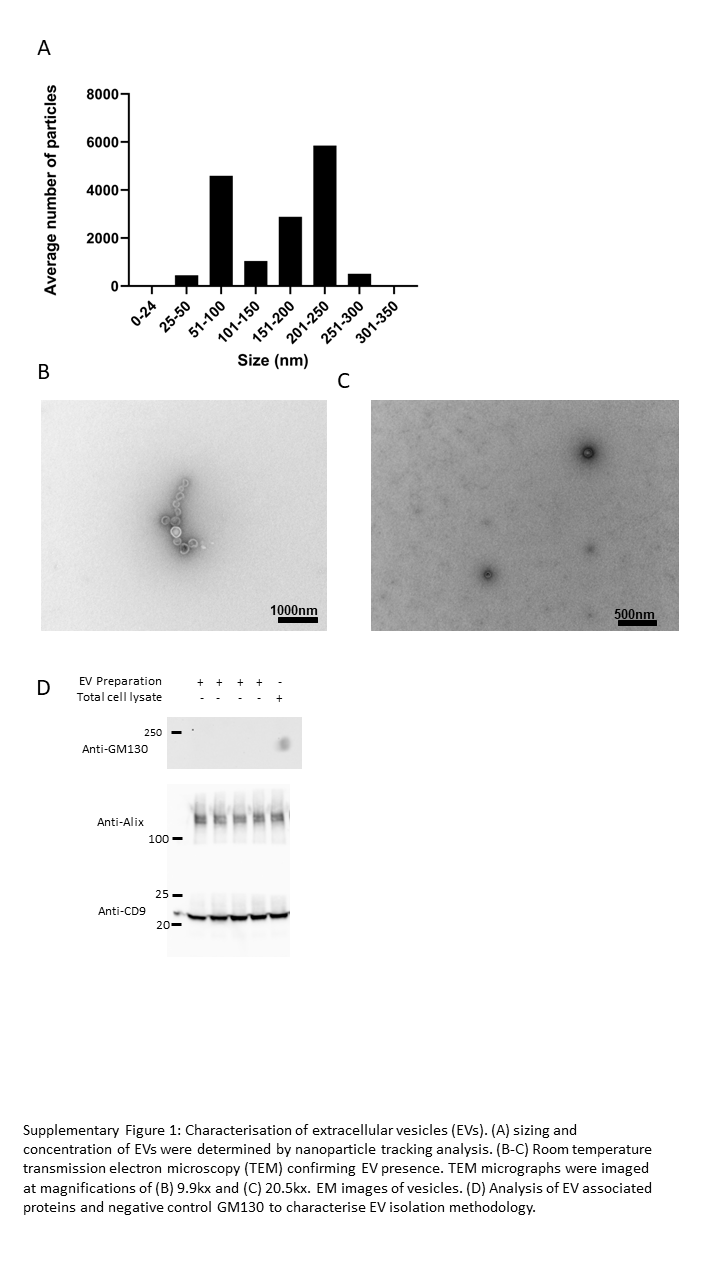

Supplement: Supplementary file 1 [file Image2.TIF]

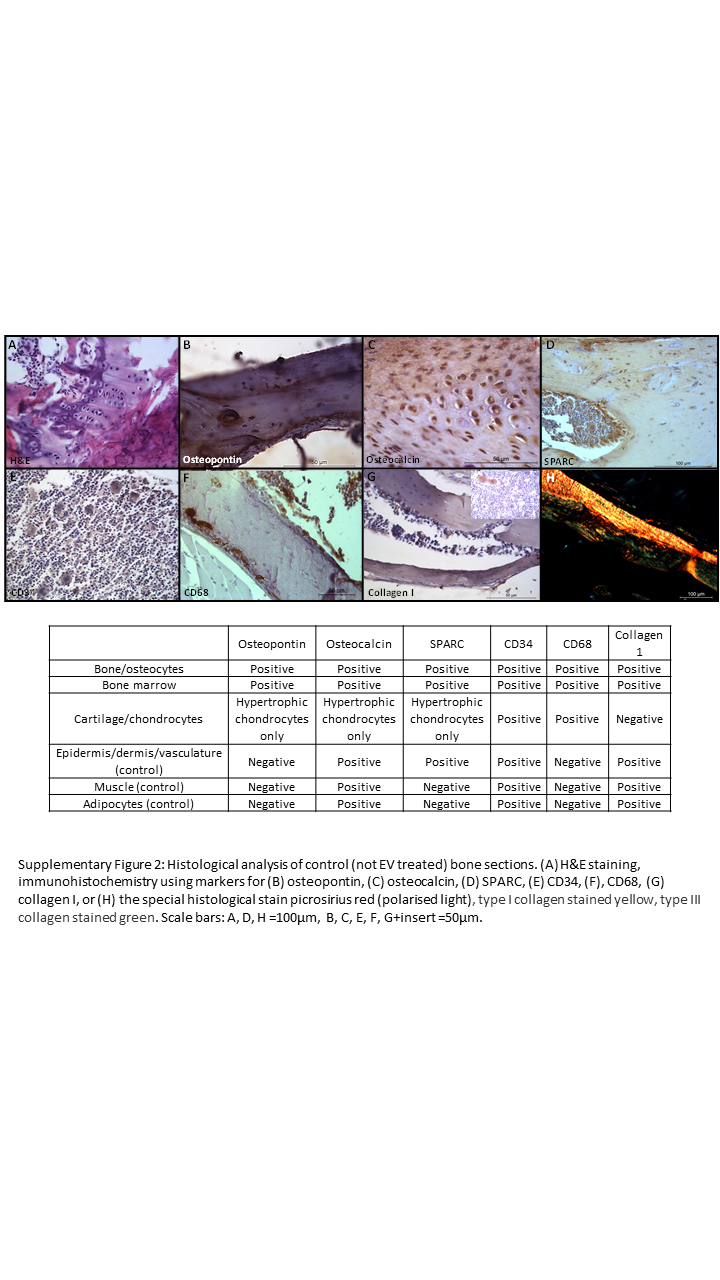

Supplement: Supplementary file 2 [file Image1.TIF]
